# Variation in and deviation from protective ventilation during lung surgery

**DOI:** 10.1016/j.xjtc.2025.10.010

**Published:** 2025-10-28

**Authors:** Matthew C. So, Camila Machado de Souza, Gordon Buduhan, Sadeesh K. Srinathan, Lawrence Tan, Biniam Kidane

**Affiliations:** a Department of Radiology, Rady Faculty of Health Sciences, University of Manitoba, Winnipeg, Manitoba, Canada; b Department of Anesthesiology, Pain and Perioperative Medicine, University of Manitoba, Winnipeg, Manitoba, Canada; c Section of Thoracic Surgery, Rady Faculty of Health Sciences, University of Manitoba, Winnipeg, Manitoba, Canada; d CancerCare Manitoba Research Institute, Winnipeg, Canada; e Department of Physiology & Pathophysiology, University of Manitoba, Winnipeg, Canada; f Department of Biomedical Engineering, University of Manitoba, Winnipeg, Canada

**Keywords:** one-lung ventilation, barotrauma, pulmonary surgical procedures, positive-pressure respiration, tidal volume

## Abstract

**Background:**

One-lung ventilation (OLV) is used for most lung resections. Protective ventilation strategies aim to limit volutrauma, atelectrauma, and barotrauma to reduce postoperative pulmonary complications (PPCs). We aimed to describe patterns in ventilatory strategies used during OLV, identify factors that affect these strategies, and investigate their relationship with PPCs.

**Methods:**

Consecutive eligible patients undergoing lung surgery at a tertiary thoracic center were enrolled in this prospective cohort study. Real-time data capture of intraoperative ventilation parameters was performed. Complications were assessed prospectively using the validated Ottawa Thoracic Morbidity and Mortality classification system. Univariate statistics were described regarding adherence to low tidal volume (V_T_), airway pressures, and positive end-expiratory pressure (PEEP). Multivariable regression models interrogated the relationships between PPCs (outcome variable), patient factors, and ventilatory parameters (predictor variables).

**Results:**

A total of 225 patients were included. The median V_T_, PEEP, driving pressure, and plateau pressure were 6.4 mL/kg, 5 cmH_2_O, 9.5 cmH_2_O, and 15.3 cmH_2_O, respectively. The percentage of surgeries within defined protective limits (V_T_ < 5 mL/kg, PEEP ≥5 cmH_2_O, driving pressure ≤15 cmH_2_O, and plateau pressure ≤25 cmH_2_O) at least 75% of the OLV time were 7.5%, 86.7%, 67.4%, and 55.8%, respectively. An increased proportion of time with peak inspiratory pressure >25 cmH_2_O (odds ratio [OR], 3.62; 95% confidence interval [CI], 1.15-12.03; *P* = .0302) and duration of OLV (OR, 3.44; 95% CI, 1.88-6.65; *P* = .00011) were associated with PPCs.

**Conclusions:**

Adherence to lung-protective ventilation recommendations was low. Higher peak inspiratory pressure is associated with PPCs, supporting barotrauma during OLV as a culprit. This is a target for quality assessment and knowledge translation efforts.

## Graphical Abstract

**Figure:**
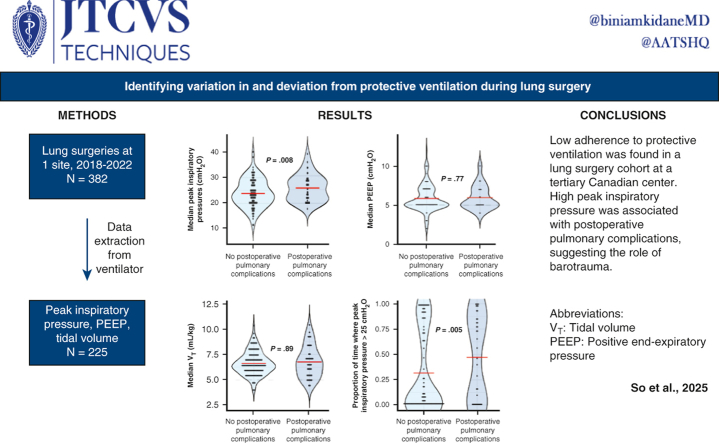

**Figure:**
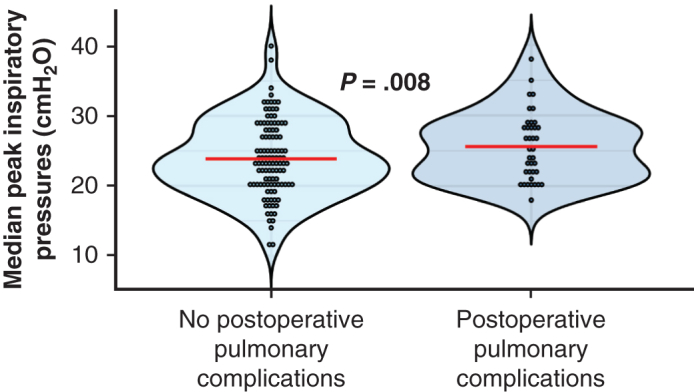
High peak inspiratory pressure was associated with postoperative pulmonary complications.

**Central Message**

Low adherence to protective ventilation was found in a lung surgery cohort. High peak inspiratory pressure was associated with postoperative pulmonary complications, suggesting the role of barotrauma.

**Perspective**

One-lung ventilation is used for most lung resections. Protective mechanical ventilation limits volutrauma, atelectrauma, and barotrauma to reduce postoperative pulmonary complications (PPCs). In a tertiary thoracic surgery center, low adherence to protective ventilation was found, and PPCs were associated with nonprotective airway pressures. These findings highlight the need for ongoing quality assessment.

One-lung ventilation (OLV) is used during most lung resection surgeries to isolate the operated lung (typically the nondependent lung) from the ventilated lung (typically the dependent lung), facilitating surgical exposure but also protecting the dependent lung from soiling secondary to blood and/or secretions. The routine use of OLV during most thoracic procedures can be injurious to both lungs if not managed carefully.^1^

There is an abundance of evidence suggesting that mechanical ventilation is a major contributor to lung injury and increased postoperative pulmonary complications (PPCs).^2^ We assume that a protective ventilation strategy, usually involving a combination of low tidal volume (V_T_), some degree of positive end-expiratory pressure (PEEP), and low ventilatory pressures, is potentially beneficial in minimizing PPCs. However, delivering protective ventilation to one lung can be difficult, as adequate gas exchange and tissue oxygenation must be maintained despite reduced compliance, and a tendency toward atelectasis frequently present in the dependent lung.

Lung injury is the most common cause of death after lung resection, and injurious mechanical ventilation can contribute to lung injury.^3^^,^^4^ The use of protective ventilation has been shown to reduce lung injury in other settings; however, little is known about the extent of adherence to protective mechanical ventilation in the setting of OLV, or the effects of nonadherence to protective mechanical ventilation on PPCs. We hypothesized that the local adherence to lung-protective ventilation regarding limits on PEEP, V_T_, and plateau pressure were low, and that nonadherence was associated with a lower rate of postoperative pulmonary complications. This study aimed to describe patterns in ventilatory strategies used during OLV, identify factors that affect these strategies, and investigate their relationship with PPCs.^5^

## Methods

Consecutive patients were recruited as part of a prospective observational cohort study of consecutive adult patients undergoing thoracic surgery involving lung resections and requiring OLV. That study is ongoing, but the present analysis uses data from December 2018 to December 2022. Of these, the included patients underwent surgery between April 2019 and March 2022. The Institutional Review Board of the University of Manitoba approved the study protocol and publication of data (approval H2018:334 [HS22088]; approved initially on September 17, 2018, and renewed annually). All included patients provided informed written consent for publication of their study data.

The dataset contains perioperative information, such as age, sex, presence of comorbidities, preoperative pulmonary function test (PFT) results, intraoperative vital signs, and multiple parameters of mechanical ventilation. All surgeries were performed at the Health Sciences Centre, a tertiary academic hospital in Canada that serves as the major referral center for thoracic surgery in the province; exclusion criteria for the study included age <18 years, known intraoperative bleeding with estimated blood loss >150 mL or requiring blood transfusions, and inability to provide informed consent. Intraoperative ventilation parameters were set by the anesthesiologist performing each case according to individual clinical judgment; a standardized protocol was not used. For the purpose of this study, protective ventilation during OLV was defined as V_T_ ≤ 5 mL·kg^−1^ of predicted body weight, PEEP ≥5 cmH_2_O, plateau pressure ≤25 cmH_2_O, and driving pressure ≤15 cmH_2_O.^6^, ^7^, ^8^, ^9^, ^10^, ^11^ All criteria were considered independently; that is, a case could use a nonprotective V_T_ while maintaining a protective PEEP. Predicted body weight was calculated using the following formulas^12^:

50 + 0.91 (height in centimeters – 152.4) for males.

45.5 + 0.91 (height in centimeters – 152.4) for females.

All intraoperative data were extracted directly from the anesthesia monitor and ventilator prospectively using monitoring software (iXTrend) except for plateau and driving pressures, which were calculated.

The indications for the vast majority of surgeries were lung resections for suspected lung cancer. Patients generally underwent sublobar resection if the suspected neoplasm met the following criteria: peripheral, small (≤2 cm), and node-negative.

### Perioperative Outcomes

Follow-up was accomplished perioperatively; patients were monitored prospectively for development of postoperative complications. These were recorded and graded prospectively by the clinical team (and confirmed by the local Quality Assurance Director) using the validated Ottawa Thoracic Morbidity and Mortality classification system based on the Clavien-Dindo system. Postoperative pulmonary complications were defined as atelectasis requiring bronchoscopic intervention, pneumonia, >48-hour postoperative mechanical ventilation, acute respiratory distress syndrome, respiratory-related death, new respiratory failure requiring home oxygen, air leak, pneumothorax, chylothorax, empyema, hemothorax, prolonged drainage, subcutaneous emphysema, or bronchopleural fistula. Pneumonia was associated with findings of new infiltrates on chest radiography with fever or leukopenia or leukocytosis, as well as new-onset cough, purulent sputum, or auscultation findings.

### Statistical Analysis

A data analysis and statistical plan was developed after the data were accessed. Adherence to protective ventilation was analyzed for each individual ventilatory parameter and presented using descriptive statistics (percentage of cases × percentage of time on OLV). A subanalysis of different V_T_ values (V_T_ < 8 mL·kg^−1^ and V_T_ < 6 mL·kg^−1^) was performed as well.

**Figure 1:**
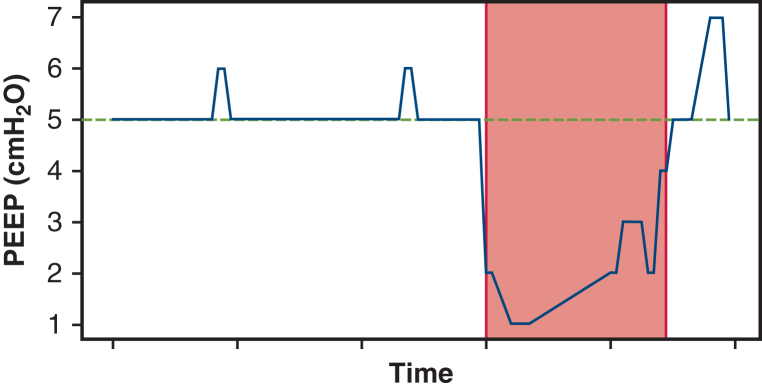
Visual representation of the “proportion of nonprotective time” predictors. In this example, the *green line* represents the cutoff for protective positive end-expiratory pressure (PEEP) of 5 cmH_2_O. Any PEEP <5 cmH_2_O (ie, below the *green line*) is considered “nonprotective” time. In this case, the value of the predictor “proportion of time spent <5 cmH_2_O” is equal to the “nonprotective time” divided by the total one-lung ventilation (OLV) time.

The associations between patient-specific and intraoperative variables with PPCs were calculated by logistic regression in R (R Foundation for Statistical Computing). Common predictors for all analyses included age, body mass index, sex, preoperative FEV1, preoperative DLCO, and duration of OLV. We did not control for the effect of any individual anesthesiologist, because multivariable models could not reliably support a covariate with 46 categories (ie, 46 unique anesthesiologists). For PPC model 1, the predictors included the common predictors as well as the amount of lung removed and median V_T_, peak inspiratory pressure, and PEEP during the OLV time. The amount of lung removed was encoded as a dummy-coded 3-level categorical variable the amount of lung removed, with the following groups: wedge/segment/biopsy/no lung removed, single lobectomy with or without wedge/segmentectomy, and bilobectomy or pneumonectomy. For PPC model 2, the common predictors and the proportion of nonprotective OLV time with V_T_ > 5 mL·kg^−1^, PEEP < 5 mL·kg^−1^, and peak inspiratory pressure >25 cmH_2_O were used. Peak inspiratory pressure <25 cmH_2_O was selected because it implies plateau pressure <25 cmH_2_O in the absence of muscular contraction. Additionally, the associations between patient-specific and intraoperative variables with metrics regarding adherence to protective ventilation were determined by linear regression in R. The outcome variables for these analyses included the median peak inspiratory pressure, PEEP, V_T_, driving pressure, and plateau pressure. The coefficient estimates, unadjusted *P* values for the slope of each coefficient estimate, and results of the *F* test for model significance were computed for each model. Figure 1 shows a representative visual illustration of how the proportion of protective time variables were calculated for model 2. The primary endpoints were the overall adherence to protective ventilation and the incidence of PPCs. The effect of confounders was controlled with multiple logistic regression for PPC analysis. No subgroups or interactions were analyzed. Any patient with missing data for a particular analysis was dropped from the analysis. An unadjusted Mann-Whitney *U*-test analysis using G∗Power with an effect size of 0.5 (considered moderate; this would be conservative for ORs of approximately 0.5 for adequate PEEP, driving pressure, and V_T_ reported in the literature) and an event rate of 0.25 would have a required sample size of 140 to obtain a power of 0.8, which is within the sample size of this study. A power calculation was not done for this analysis a priori. An accurate power analysis for the primary analysis (multivariable logistic regression) would have been difficult, as much of the literature (especially that on randomized trials) focuses on comparisons between 2 groups (eg, low PEEP vs high PEEP), and the distributions of the underlying predictor variables would not have been known a priori. Therefore, the completed power analysis used the Mann-Whitney *U*-test in G∗Power with an assumed moderate effect size. For the Central Picture and Graphical Abstract only, statistical results were compared between the PPC and non-PPC groups using the Mann-Whitney *U*-test.

A full description of the data collection and preprocessing methodology and unadjusted statistical methodology is provided in the Appendix E1.

## Results

Between December 2018 and December 2022, data from 382 patients undergoing lung resection surgeries under OLV were collected prospectively collected. The indication for surgery was suspected lung cancer in the large majority of patients (n = 366) and diagnostic wedge resections for interstitial lung disease in the remaining 16. Complete ventilatory data were available for 225 patients. The majority of the missing ventilatory data was from the acute phases of COVID-19, during which intraoperative data collection of ventilatory parameters by research staff was not possible or was not allowed owing to safety concerns. Outcome data were missing for 15 patients owing to delays in data entry. Regarding adherence analyses, 43 patients received volume-controlled ventilation (VCV) and met the criteria for analysis of plateau and driving pressures. Regarding PPC analyses, 155 patients had complete data for all covariates. Table 1 summarizes patient characteristics. Sublobar resections (n = 132) comprised the bulk of all resections and included 120 wedge resections. Lobectomies (n = 89), bilobectomies (n = 2), and pneumonectomies (n = 2) composed the remaining surgeries performed in this cohort. No patient had received a pneumotoxic agent such as bleomycin, neoadjuvant therapy, or oropharyngeal or neck radiation. Video-assisted thoracoscopic surgery comprised the vast majority of procedures (n = 206), with only 17 open procedures performed.

**Table 1:** Patient characteristics

Characteristic	Value	Missing, n
Age, y, median (IQR)	67.5 (61-74)	0
Female sex, %	52.0	11
Smoking status, pack-years, median (IQR)	20.0 (2-39)	51
BMI, kg·m^−2^, median (IQR)	28.3 (25-32)	22
DLCO, % predicted, median (IQR)	81.0 (68-94)	21
FEV1, % predicted, median (IQR)	88.0 (76-106)	25
FVC, % predicted, median (IQR)	95.0 (83-108)	5
FEV_1_/FVC, % predicted, median (IQR)	95.5 (86-102)	0
Obstructive pattern PFT, %∗	15.0	25
Restrictive pattern PFT, %†	15.5	25
Mixed pattern PFT, %‡	5.0	25
PPC incidence, %	23.4	15
Total N	225	

Dichotomous values are reported as a fraction of the total number of patients for which data is nonmissing.

*IQR*, Interquartile range; *BMI*, body mass index; *DLCO*, diffusion capacity of the lung for CO; *FEV1*, forced expiratory volume in 1 second; *FVC*, forced vital capacity; *PFT*, pulmonary function test; *PPC*, postoperative pulmonary complication.

∗ Obstructive pattern PFT defined as FEV_1_/FVC <85% predicted and FVC >80% predicted.

† Restrictive pattern PFT defined as FVC <80% predicted and FEV_1_/FVC ≥85% predicted.

‡ Mixed pattern PFT defined as FEV_1_/FVC < 85% predicted and FVC <80% predicted.

Adherence to protective ventilation was heterogeneous for the various parameters, with PEEP ≥5 cmH_2_O the most frequently used ventilatory setting within protective targets. A protective V_T_ of <5 mL·kg^−1^ was used infrequently; only 5% of procedures met this protective target range for >90% of the time on OLV (Table 2). All patients were ventilated with some level of PEEP, and cases of 0 PEEP were absent in our sample.

**Table 2:** Adherence to protective ventilation

Parameter	Protective >50% of OLV duration	Protective >75% of OLV duration	Protective >90% of OLV duration
V_T_ ≤ 5 mL·kg^−1^, % (n/N)	11.2 (24/214)	7.5 (16/214)	4.7 (10/214)
V_T_ ≤ 6 mL·kg^−1^, % (n/N)	36.4 (78/214)	25.7 (55/214)	20.1 (43/214)
V_T_ ≤ 8 mL·kg^−1^, % (n/N)	84.6 (181/214)	75.7 (162/214)	66.8 (143/214)
PEEP ≥5 cmH_2_O, % (n/N)	90.7 (204/225)	86.7 (195/225)	74.2 (167/225)
Plateau pressure ≤25 cmH_2_O, % (n/N)	95.3 (41/43)	67.4 (29/43)	46.5 (20/43)
Driving pressure ≤15 cmH_2_O, % (n/N)	88.4 (38/43)	55.8 (24/43)	32.6 (14/43)

Each cell in the table represents the proportion of the total number of procedures for which data are available for which a particular ventilatory parameter (rows) was within defined protective limits for a certain proportion of time on one-lung ventilation (OLV) (columns). The defined protective limit for tidal volume (V_T_) in this study was ≤5 mL·kg^−1^; however, as a sensitivity analysis, other cutoffs in the literature were analyzed as well.

*PEEP*, Positive end-expiratory pressure.

**Figure E1:**
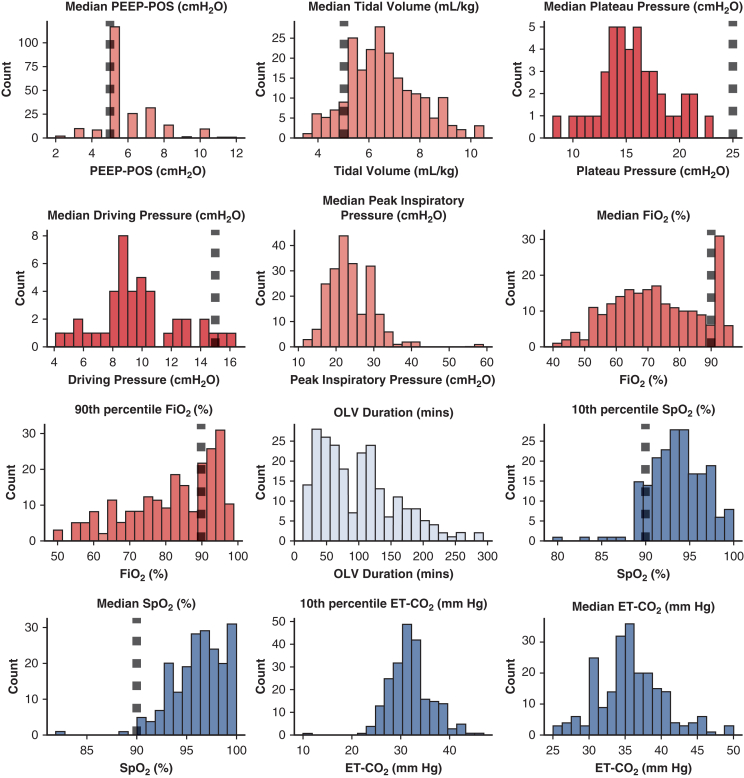
Histograms for selected intrasurgical vital and ventilatory parameters. *The black dotted lines* represent previously defined limits for protective ventilation, where applicable. Cutoffs: positive end-expiratory pressure (PEEP), ≥5 cmH_2_O; tidal volume (VT), ≤5 mL/kg; plateau pressure, ≤25 cmH_2_O; driving pressure, ≤15 cmH_2_O. Additionally, cutoffs for peripheral oxygen saturation (SpO_2_) >90% and fraction of inspired oxygen (FiO_2_) <90% are shown here for clarity purposes but are not analyzed elsewhere.

The median V_T_ was 6.4 mL·kg^−1^ (IQR, 5.6-7.3 mL·kg^−1^), the median PEEP was 5 cmH_2_O (IQR, 5-7 cmH_2_O), the median estimated plateau pressure was 15.3 cmH_2_O (IQR, 13.7-17.6 cmH_2_O), and the median driving pressure was 9.5 cmH_2_O (IQR, 8.3-10.8 cmH_2_O). The median peak inspiratory pressure was 23 cmH_2_O (IQR, 20-28 cmH_2_O). Histograms describing the data distribution of ventilatory parameters are separately visualized (Figure E1).

**Figure 2:**
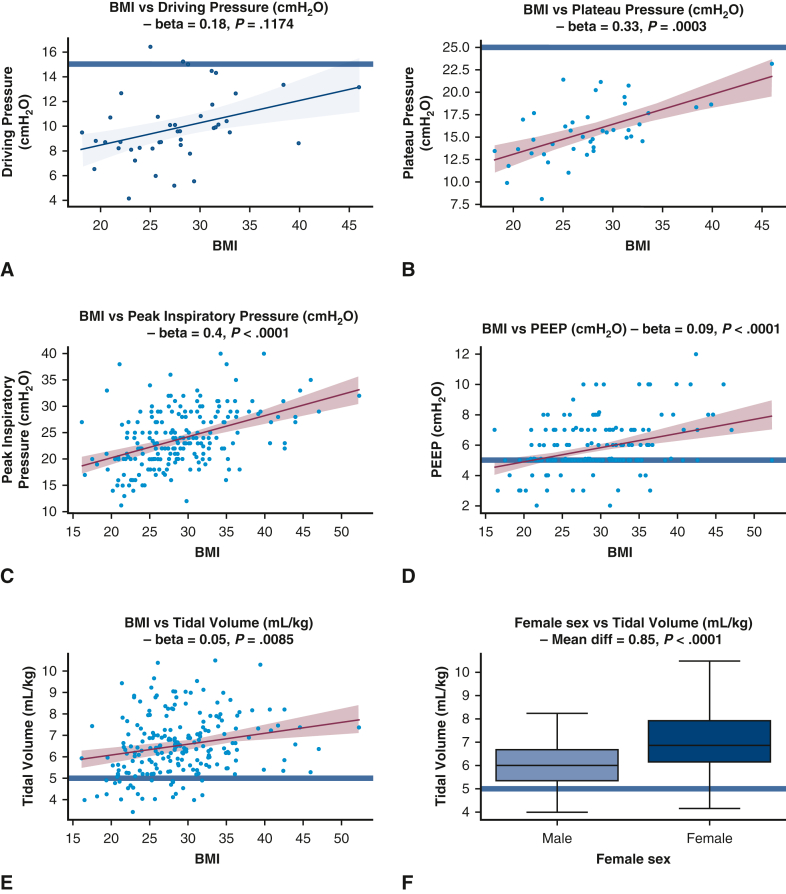

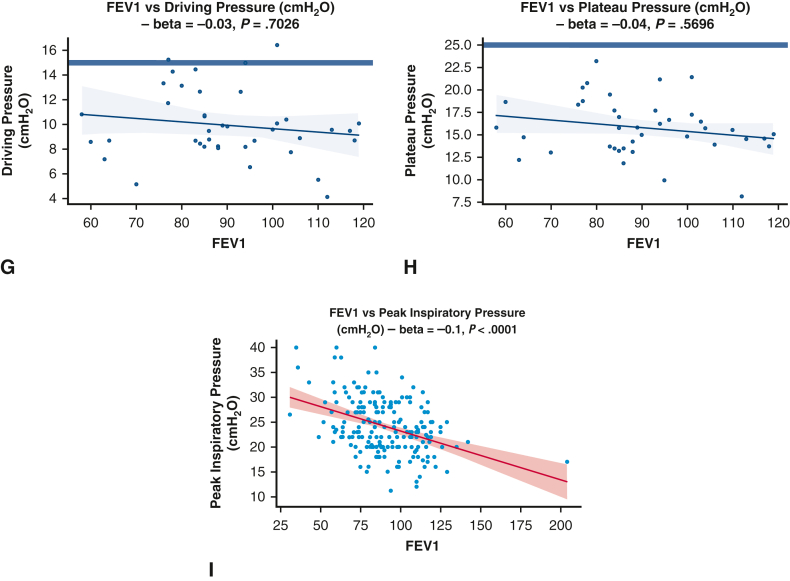
Unadjusted analyses comparing presurgical predictors with median ventilatory parameters. A, Scatterplot showing the relationship between body mass index (BMI) and driving pressure, not statistically significant. B-E, Scatterplots showing a significant positive association between BMI and plateau pressure (B), between BMI and peak inspiratory pressure (C), between BMI and positive end-expiratory pressure PEEP (D), and between BMI and tidal volume (E). F, Boxplot showing a significant increase in tidal volume with female sex. G and H, Scatterplots showing a nonsignificant negative association between FEV_1_ and driving pressure (G) and between forced expiratory volume in 1 second (FEV1) and plateau pressure (H). I, Scatterplot showing a significant positive association between FEV1 and peak inspiratory pressure. *Blue horizontal lines* represent the boundary for defined protective limits: tidal volume, ≤5 mL/kg; positive end-expiratory pressure (PEEP), ≥5 cmH_2_O; plateau pressure, ≤25 cmH_2_O; driving pressure, ≤15 cmH_2_O. Each *blue dot* represents 1 subject, and each trendline shows the best-fit univariate linear regression and associated confidence interval. Colored analyses represent analyses that are statistically significant at *P* < .05. *BMI*, Body mass index, *FEV1*, forced expiratory volume in 1 second.

All ventilatory parameters (V_T_, PEEP, plateau pressure, and driving pressure) were significantly higher during OLV in obese patients (body mass index >30 kg m^−2^), as demonstrated by the generalized linear model analyses and univariate analyses (Tables 3 and 4, Figure 2). The median V_T_ used during OLV was 6.67 mL·kg^−1^ in obese patients versus 6.28 mL·kg^−1^ in nonobese patients (*P* = .03). Despite this increase, plateau and driving pressures were generally maintained within protective ranges (Figure 2).

**Table 3:** Factors associated with variations in specific ventilatory parameters

Factor	(a) Peak inspiratory pressure	(a) *P* value	(b) PEEP	(b) *P* value	(c) V_T_	(c) *P* value
Age	−0.04 [−0.11 to 0.03]	.26	−0.02 [−0.04 to 0]	.08	0.01 [−0.01 to 0.02]	.51
Female sex	−0.6 [−2.04 to 0.85]	.42	−0.38 [−0.85 to 0.09]	.12	0.99 [0.62-1.36]	**<.0001**
BMI	0.4 [0.27-0.53]	**<.0001**	0.1 [0.05-0.14]	**<.0001**	0.05 [0.02-0.09]	**.0019**
OLV duration	−0.23 [−1 to 0.54]	.55	0.22 [−0.03 to 0.46]	.083	0.1 [−0.1 to 0.29]	.32
FEV1, % predicted	−0.09 [−0.13 to −0.05]	**<.0001**	0 [−0.01 to 0.02]	.7	0 [−0.01 to 0.01]	.63
DLCO, % predicted	0 [−0.04 to 0.05]	.87	0 [−0.01 to 0.02]	.92	0 [−0.01 to 0.02]	.64
Model	N = 164	**<.0001**	N = 172	**<.0001**	N = 172	**<.00001**

Each pair of columns shows the coefficients of a separate linear regression model regressing predictive factors—age, sex, body mass index (BMI), one-lung ventilation (OLV) duration, forced expiratory volume in 1 second (FEV1), diffusion capacity of the lung for CO (DLCO)—against the median intrasurgical (a) peak inspiratory pressure, (b) positive end-expiratory pressure (PEEP), (c) tidal volume (V_T_). In total, 5 linear regression models were fitted in this analysis. The leftmost column of each pair shows the linear regression coefficient, while the rightmost column of each pair shows the *P* value for the significance of this predictor. For example, greater BMI is associated with higher median peak inspiratory pressure, PEEP, V_T_, plateau pressure, and driving pressure, while a lower FEV1 is significantly associated only with a higher peak inspiratory pressure. The bottom-most row shows the sample size and *P* value comparing the model's explanatory power to a null model using analysis of variance. Bold type indicates statistical significance at *P* < .05.

**Table 4:** Factors associated with PPCs using adjusted analysis

Parameter	Model 1 (median V_T_, PIP, PEEP), OR (95% CI)	*P* value	Model 2 (proportion nonprotective V_T_^1^, PIP,^2^ PEEP^3^), OR (95% CI)	*P* value
OLV duration	3.48 (1.92-6.69)	**<.0001**	3.44 (1.88-6.65)	**.00011**
BMI	0.95 (0.86-1.04)	.27	0.98 (0.9-1.08)	.73
Female sex	0.66 (0.26-1.69)	.39	0.89 (0.36-2.27)	.81
Age	1.01 (0.97-1.06)	.64	1.02 (0.98-1.08)	.37
FEV1 (% predicted)	0.99 (0.97-1.02)	.67	1 (0.97-1.02)	.76
DLCO (% predicted)	0.98 (0.95-1.01)	.14	0.97 (0.94-1)	.0882
Surgery, wedge/segmentectomy	Reference category			
Surgery, lobectomy	0.49 (0.15-1.52)	.22	0.7 (0.21-2.21)	.54
Surgery, bilobectomy or pneumonectomy	1.15 (0.07-34.42)	.92	1.64 (0.07-68.73)	.77
V_T_	1.05 (0.74-1.48)	.79	0.16 (0.02-1.19)	.0745
Peak inspiratory pressure	1.1 (1-1.22)	.0505	3.62 (1.15-12.03)	**.0302**
PEEP	0.97 (0.73-1.28)	.85	0.31 (0.04-1.82)	.22
Overall model	N = 155	**<.0001**	N = 155	**<.0001**

Each pair of columns represents the odds ratios (ORs), with 95% confidence intervals (CIs), from a logistic regression model fitted to predict the outcome variable of postoperative pulmonary complications (PPCs). The predictor variables are shown in the leftmost column, including one-lung ventilation (OLV) duration, body mass index (BMI), female sex, age, forced expiratory volume in 1 second (FEV1), diffusion capacity of the lung for CO (DLCO) for both models, plus the following variables: in model 1, the median intrasurgical tidal volume (V_T_), peak inspiratory pressure, and peak end-expiratory pressure (PEEP); in model 2, (1) proportion of OLV time spent with VT > 5 mL·kg^−1^, (2) proportion of OLV time spent with peak inspiratory pressure >25 cmH_2_O, and (3) proportion of OLV time spent with PEEP <5 cmH_2_O. The leftmost column of each pair shows the OR, while the rightmost column of each pair shows the *P* value for the significance of this predictor. In model 2, higher proportion of nonprotective time implies higher peak inspiratory pressure, higher V_T_, and lower PEEP. The bottom-most row shows the sample size and *P* value comparing the model's explanatory power to a null model using analysis of variance. Bold type indicates statistical significance at *P* < .05.

Female sex also was associated with higher median V_T_ during OLV compared to males (unadjusted analysis: 6.87 mL·kg^−1^ vs 5.99 mL·kg^−1^, *P* < .0001; adjusted analysis: 0.99 [IQR, 0.62-1.36], *P* < .0001). Peak inspiratory pressures and plateau pressures were higher in patients with restrictive lung disease based on the preoperative PFTs (Figure 2).

The incidence of hypoxemia (SpO_2_ <90%) for >60 seconds during OLV was 28%. The median total duration of hypoxemia during OLV was 208.5 seconds (IQR, 63.25-608.25 seconds) and the median fraction of inspired O_2_ (FiO_2_) used was 72% (IQR, 62%-85%). The median V_T_ was not significantly different in patients with and those without >60 seconds of hypoxemia during OLV (median V_T_, 6.48 mL/kg in hypoxemia and 6.35 mL/kg without hypoxemia; *P* = .578). The median duration of OLV was 88 minutes (IQR, 50-129 minutes).

Increased OLV duration in hours (adjusted OR, 3.44; 95% CI, 1.88-6.65, *P* < .001 with n = 155; unadjusted OR, 3.48; 95% CI, 1.92-6.69, *P* < .001 with n = 210) and an increased proportion of OLV time with peak inspiratory pressure >25 cmH_2_O (adjusted OR, 3.62; 95% CI, 1.15-12.03; *P* = .0302; unadjusted OR, 3.16; 95% CI, 1.45-6.93; *P* = .0038) were associated with an increased rate of PPCs after adjusting for demographic variables, surgery type, and preoperative FEV_1_ and DLCO. Additionally, in unadjusted analyses, greater median peak inspiratory pressure was associated with an increased risk of complications (adjusted OR, 1.1; 95% CI, 1-1.22, *P* = .0505 with n = 155; unadjusted OR, 1.06; 95% CI, 1.01-1.13, *P* = .0275 with n = 202) (Table 4). Additionally, in unadjusted analyses, both FEV1 (OR, 0.98; 95% CI, 0.96-1; *P* = .0214) and DLCO (OR, 0.97; 95% CI, 0.95-0.99; *P* = .0149) were associated with PPCs.

## Discussion

Our study demonstrates that adherence to protective ventilatory strategies during OLV is low in this population of patients undergoing lung resection surgery, as evidenced by the reduced proportion of patients with low V_T_, low plateau pressure, and low driving pressure for >90% of the time on OLV. Previous observational studies have identified similar results in the general and thoracic surgery population.^11^^,^^13^ The evidence supporting the role of protective ventilation in reducing PPCs has produced conflicting results, and there is a paucity of randomized trials focused on OLV.^6^^,^^11^^,^^14^, ^15^, ^16^ This may explain why anesthesia providers may be slow to incorporate some of the recommendations related to protective mechanical ventilation into clinical practice, as reflected by the low adherence demonstrated in this study. Lung-protective ventilation during OLV also can be clinically difficult, given the numerous competing priorities in these cases, and patients needing lung surgery often have compromised lung function due to the insult (eg, smoking, interstitial lung disease) that led to the need for lung surgery. Attempting to maintain oxygenation and ventilation while keeping V_T_, pressures, and FiO_2_ low during OLV in this setting is especially challenging at the ground level.

Of all the components of this package termed protective ventilation, maintaining small V_T_ during OLV appeared to be most difficult to achieve. For the purpose of this study, we defined 5 mL·kg^−1^ as protective V_T_, recognizing that there is no consensus on the ideal V_T_ for OLV.^8^^,^^17^ Maintaining a large VT was once considered standard of care, when the primary concern during OLV was to prevent hypoxemia and avoid atelectasis; however, hyperinflation induced by large V_T_ (volutrauma) is a well-described mechanism of ventilator-induced lung injury,^2^ and there is substantial evidence recommending against this practice, especially during OLV.^2^^,^^15^ In our cohort, only 67% of patients received V_T_ < 8 mL/kg of predicted body weight for the majority of the time on OLV. This represents a significant deviation from the recommended V_T_ during OLV.^17^^,^^18^ One possible explanation for this may simply reflect a failure to adjust ventilation based on predicted instead of actual body weight. This would have a particularly relevant impact in patients who are overweight and female patients, considering the usual equations used in calculating predicted body weight. It also would explain why in our sample, obesity and female sex were risk factors for receiving higher than optimal V_T_ during mechanical ventilation.

Barotrauma and volutrauma play major roles in ventilator-induced lung injury.^2^^,^^19^ They are inextricably linked, and overdistension of alveoli leads to inflammation and vascular permeability.^2^^,^^19^ Barotrauma is associated with regional lung overdistension, related to air leak and pneumothorax. Atelectrauma, the continuous opening and closure of alveoli during each respiratory cycle, is also injurious to the lungs.^2^^,^^19^ Thus, avoiding lung collapse and excessive atelectasis during mechanical ventilation is an important aspect of protective ventilation and becomes particularly relevant when reduced V_T_ is used.^20^^,^^21^

PEEP during OLV improves oxygenation and minimizes lung injury.^6^^,^^19^ All our patients were ventilated with some degree of PEEP, and we found no cases of 0 PEEP, consistent with recommendations made by experts and guidelines.^6^^,^^19^ However, clearly we did not make adequate use of the full benefits of PEEP titration during OLV, considering that only 70% of our patients received >5 cmH_2_O of PEEP for a majority of their OLV time. This may explain the much higher than expected incidence of hypoxemia during OLV in our patients.^20^ Competing demands and the need to oxygenate patients might have led to higher peak inspiratory pressure and greater use of recruitment maneuvers in these patients, both of which are associated with a higher rate of PPCs, further exacerbated by the greater baseline risk of developing PPCs in this patient population.^22^

Lower FEV_1_ and DLCO are known risk factors for PPCs. In our cohort, we also identified this association on unadjusted analyses, but not on adjusted analyses. A possible explanation is that intraoperative ventilation parameters are the primary drivers of PPCs independent of pulmonary function testing. Additionally, our practice avoids operating on patients with prohibitively poor PFTs or uses sublobar resections in those with borderline PFTs.

The ideal PEEP for OLV is not well defined, but evidence suggests it is likely >5 cmH_2_O. Multiple studies have clearly demonstrated improved oxygenation and lung mechanics during OLV with PEEP levels >5 cmH_2_O: 10 ± 2 cmH_2_O in the study by Ferrando and colleagues^23^ and 8.1 ± 2.1 cmH_2_O in the recently published i-PROVE-OLV.^6^ Spadaro and colleagues^24^ also reported a significant and more pronounced reduction in shunt fraction during OLV when PEEP was titrated to 10 cmH_2_O instead of 5 cmH_2_O. These findings were corroborated by a recent experimental study demonstrating that PEEP up to 15 cmH_2_O did not increase intrapulmonary shunt during OLV.^25^ Optimizing driving pressure also improves oxygenation and lung mechanics during OLV,^16^^,^^25^ and the lack of adherence to low driving pressures also might have contributed to the high incidence of hypoxemia in our sample.

To the best of our knowledge, plateau pressure has not been studied in isolation during OLV. However, most studies of protective ventilation during OLV have reported on this and made the point of limiting plateau pressures as part of the method to avoid barotrauma, as supported by literature from other settings.^6^^,^^16^ Again, providers in our sample were unable to maintain plateau pressures within protective limits for a significant number of patients for the majority of time on OLV. The same can be said for peak airway pressures, which were significantly elevated during OLV in our cohort. Peak airway pressures are typically higher than the pressure applied to the alveoli because of the large component of the airflow resistance generated by the endotracheal tube, which is narrower during OLV. However, if peak airway pressures are high, then most likely the risk of barotrauma is increased. This is corroborated by our finding of a higher rate of PPCs the longer patients are exposed to peak inspiratory pressure >25 cmH_2_O during OLV. Exposure to OLV in itself is also a major risk factor for PPCs, as is well documented in the literature and again demonstrated in the present study.

This study has a number of limitations. Plateau pressures were not collected as part of our original dataset, and thus we devised a method of estimating plateau pressure based on the pressure and flow curves of patients who received VCV. A minimal inspiratory pause of 10% at the end of inspiration is delivered as default by our operating room ventilators during VCV mode, but not in pressure-controlled ventilation mode. This pause might not have been long enough to allow for pressure to equilibrate throughout the lungs. In other words, the pressures that we measured likely overestimated the real plateau pressures in our sample, but the magnitude of this overestimation is uncertain. This method also may have affected our estimation of driving pressure to some extent. The sample for this study was derived from a single site, which may limit the generalizability of our data to other populations, especially those with a lower incidence of obesity. Another limitation was our inability to control for anesthesiologist provider-level effects.

Limitations notwithstanding, our findings convey an important message that when there is this degree of variation, divorced from the effect of individual anesthesiologists, there can be lower levels of adherence to challenging guidelines at the system level, and that this can have deleterious/harmful consequences. The finding of an apparent low degree of adherence to protective V_T_ parameters in our population may appear to be a limitation; however, from a scientific perspective it is a strength, as it represents a real-world assessment of adherence in a high-volume tertiary thoracic surgery center and thus facilitates assessment of the potential sequelae of variations in adherence to lung-protective ventilation parameters during OLV.

## Conclusions

OLV is commonly used in lung resection surgeries to facilitate the operation and protect the ventilated lung but can be potentially harmful if not carefully managed. Protective mechanical ventilation strategies have been described to minimize the risk of ventilator-induced injuries. At a major tertiary thoracic surgery center, we demonstrated very low adherence to protective ventilation during OLV, as evidenced by the reduced proportion of patients who received a low V_T_, low plateau pressure, and low driving pressure for >90% of the time on OLV. A higher proportion of OLV time with peak inspiratory pressure >25 cmH_2_O and increased OLV duration were associated with an increased rate of PPCs.

Despite being about anesthetic exposures and management, our findings are especially relevant to thoracic surgeons. The sources of perioperative risk are highly varied, certainly including surgical technique but also including patient selection, postoperative care, and anesthetic exposures/technique. Multidisciplinary collaboration including surgeons has helped improve outcomes in lung surgery across multiple domains.

Thoracic surgeons have worked hard to improve outcomes of lung surgery by identifying the sources of risk and understanding variability across centers as well as between high-performing and low-performing centers, using such databases STS, ESTS, NSQIP, and others. We have come to understand that many (if not most) risk factors are not actually related to surgical technique; for example, lack of antibiotic and venous thromboembolism prophylaxis is a known risk factor for the development of complications that are not technical and could be argued to be of less interest to surgeons. However, through study and acknowledgment that these factors impact our lung surgery outcomes, the thoracic surgical community has come to see them as important and relevant to surgeons. Surgeons have spearheaded quality improvement in these traditional “nonsurgical” domains to ensure better outcomes for our lung surgery patients. Intraoperative anesthetic risk factors are real and important. The problem is that they are generally a “black box” for surgeons and are unclear and underreported and understudied. For thoracic surgeons to continue to improve outcomes for lung surgery patients, we need to understand whether there is a risk hiding in plain sight that may be more important than many of the risk factors we are constantly chasing and trying to incrementally improve (eg, VATS vs RATS, uniport vs multiport, etc). The fundamental issue remains how to fix something that we do not understand or even know is a problem. By necessity, this issue of anesthetic exposures/management must become more salient to thoracic surgeons so that we can start working with our anesthesia colleagues to implement quality improvement in this domain.

Further validation and mechanistic work is underway to understand the mechanisms through omics-level work (ie, proteomics, lipidomics, metabolomics). Larger, multicenter studies are also planned to further externally validate this work. This information may help minimize the risk of complications after lung resections requiring OLV. From a knowledge translation perspective, we plan to use these data to further investigate barriers to increasing adherence.

## Conflict of Interest Statement

The authors reported no conflicts of interest.

The *Journal* policy requires editors and reviewers to disclose conflicts of interest and to decline handling or reviewing manuscripts for which they may have a conflict of interest. The editors and reviewers of this article have no conflicts of interest.

## Appendix E1

Whenever VCV is used, a minimal inspiratory pause of 10% at the end of inspiration is delivered as default by the ventilator available in our operating rooms. Plateau pressures were computed from the pressure–time and flow–time curves. The pressure and flow curves were initially resampled to 50 Hz and smoothed using a Savitsky-Golay filter. Only cases in which VCV was used for at least 50% of detected breaths were considered for the analysis of plateau and driving pressures. The plateau pressure for each breath was set at a point on the pressure curve where the smoothed flow crossed from a positive value to a negative value prior to the point of maximal negative flow. The effect of this algorithm was to select the last point at the end of the inspiratory pause at which flow was approximately equal to 0, thereby selecting the plateau period. The resulting plateau pressure was then resampled to 1 Hz. The driving pressure was calculated using the difference between the computed plateau pressure and the PEEP recorded from the ventilator. All data preprocessing was performed using Python.

All ventilatory data were resampled to 1 Hz prior to analysis. Ventilatory parameter targets were set by the anesthesiologist on the ventilator, which attempted to maintain these levels for the ventilated lung. For example, a PEEP target of 5 cmH_2_O was most often set; however, the actual level of PEEP delivered (on a per-second basis) was analyzed in the study. Targets for plateau pressure (in cmH_2_O) and tidal volume (in mL) were set similarly.

Patients with <10 minutes of recorded monitoring data during OLV and patients missing data on all of ventilatory parameters (V_T_, PEEP, and peak inspiratory pressure) were removed from the analysis. Otherwise, all available data were analyzed for each patient; that is, a missing PEEP value did not affect the analysis of V_T_, and so on.

Selected predictors considered to affect ventilatory parameters were further investigated in an unadjusted analysis. For this analysis, an obstructive PFT pattern was defined as FEV_1_/FVC <85% predicted and FVC >80% predicted, a restrictive PFT pattern was defined as FEV_1_/FVC ≥ 85% predicted and FVC < 80% predicted, and a mixed PFT pattern was defined as FEV_1_/FVC <85% predicted and FVC <80% predicted. A 2-sided independent-samples *t* test was used to compute the *P* value for a statistically significant difference in group means. For the unadjusted analysis only, *P* values were adjusted for multiple comparisons using the Holm-Sidak method.

Histograms were used to visualize the statistical distribution of ventilatory parameters in this dataset. Each count refers to a single surgery, which was summarized with the median, 10th percentile, or 90th percentile for each ventilatory variable (Figure E1). The OLV duration distribution was also plotted.
